# Sequencing of *bla*_IMP_-Carrying IncN2 Plasmids, and Comparative Genomics of IncN2 Plasmids Harboring Class 1 Integrons

**DOI:** 10.3389/fcimb.2017.00102

**Published:** 2017-03-30

**Authors:** Xiaoyuan Jiang, Zhe Yin, Xiuyun Yin, Haihong Fang, Qiang Sun, Yigang Tong, Yuanhong Xu, Defu Zhang, Jiao Feng, Weijun Chen, Yajun Song, Jinglin Wang, Shuiping Chen, Dongsheng Zhou

**Affiliations:** ^1^Anhui Medical UniversityHefei, China; ^2^State Key Laboratory of Pathogen and Biosecurity, Beijing Institute of Microbiology and EpidemiologyBeijing, China; ^3^Department of Clinical Laboratory, The 307th Hospital of the People's Liberation ArmyBeijing, China; ^4^Department of Clinical Laboratory, The First Affiliated Hospital of Anhui Medical UniversityHefei, China; ^5^College of Food Science and Project Engineering, Bohai UniversityJinzhou, China; ^6^Beijing Institute of Genomics, Chinese Academy of SciencesBeijing, China

**Keywords:** IncN2 plasmids, class 1 integron, transposon, *bla*_IMP_, antimicrobial resistance

## Abstract

This work presents the complete nucleotide sequences of p0801-IMP from *Klebsiella pneumoniae*, p7121-IMP from *K. oxytoca*, and p17285-IMP from *Citrobacter freundii*, which are recovered from three different cases of nosocomial infection. These three plasmids represent the first fully sequenced *bla*_IMP_-carrying IncN2 plasmids. Further comparative genomics analysis of all the five integron-carrying IncN2 plasmids p0801-IMP, p7121-IMP, p17285-IMP, pJIE137, and p34983-59.134kb indicates that they possess conserved IncN2 backbones with limited genetic variations with respect to gene content and organization. Four class 1 integrons (*bla*_IMP-1_-carrying In1223 in p0801-IMP/p7121-IMP, *bla*_IMP-8_-carrying In655 in p17285-IMP, In27 in pJIE137, and In1130 in p34983-59.134kb), two insertion sequence-based transposition units (IS*Ecp1*-*orfRA1-14* in p17285-IMP, and IS*Ecp1*-*bla*_CTX-*M*-62_-Δ*orf477*-*orfRA1-14* in pJIE137), and a novel Tn*1696*-related transposon Tn*6325* carrying In1130 in p34983-59.134kb are indentified in the plasmid accessory regions. In1223 and In655 represent ancestral Tn*402*-associated integrons, while In27 and In1130 belong to complex class 1 integrons. The relatively small IncN2 backbones are able to integrate different mobile elements which carry various resistance markers, promoting the accumulation and spread of antimicrobial resistance genes among enterobacterial species.

## Introduction

The Ambler B metallo-β-lactamases IMPs are capable of hydrolyzing almost all β-lactams including carbapenems and, to date, 52 IMP-variant enzymes have been reported in at least 26 species of clinically important Gram-negative organisms such as *Pseudomonas, Acinetobacter* and *Enterobacteriaceae* species all over the world (Zhao and Hu, 2015). IMP producers often employ additional mechanisms (e.g., membrane permeability defects) and have gained significant attention due to their high-level resistance to carbapenems.

Class 1 integrons commonly carry a 5′-conserved segment [5′-CS], which is composed of the integrase gene *intI1*, a specific recombination site *attI1* located next to *intI1* and recognized by *intI1*, and a promoter Pc driving the transcription of cassette-borne genes and lying within *intI1* (Partridge et al., 2009; Domingues et al., 2012; Gillings, 2014). The *bla*_IMP_ genes are often found together with other resistance genes in the variable gene cassette arrays of class 1 integrons, and these integrons are further associated with mobile elements such as transposons and plasmids, leading to the easily mobilization of cassette-borne resistance genes across various bacterial species (Gillings et al., 2008).

Plasmids belonging to the IncN incompatibility group are the important mobile genetic platforms for dissemination of clinically important resistance genes among enterobacterial species (Poirel et al., 2011; Chen et al., 2012; Partridge et al., 2012; Netikul et al., 2014; Sun et al., 2015; Tijet et al., 2016). The IncN group can be further divided into three subgroups IncN1 to IncN3. These three subgroups have very similar backbone gene organization but with limited nucleotide sequence homology over the backbones. There is still no report of *bla*_IMP_-carrying IncN2 or IncN3 plasmid.

This work present the complete nucleotide sequences of three novel IncN2 plasmids, p0801-IMP from *Klebsiella pneumoniae*, p7121-IMP from *K. oxytoca*, and p17285-IMP from *Citrobacter freundii*. p0801-IMP and p17285-IMP harbor the class 1 integrons In1223 and In655 carrying the cassette arrays *bla*_IMP-1_-*gcu162*-*aacA4*- *aadA6* and *bla*_IMP-8_-*aacA4*, respectively. Further comparative genomics assay of all the fully sequenced integron-carrying IncN2 plasmids indicates that different mobile elements including integrons, transposons and insertion sequence-based transposition units can be inserted through transposition at different sites of the relatively small IncN2 backbones. Data presented here would promote us to gain insights into genetic variation and evolutionary history of IncN2 plasmids.

## Materials and methods

### Bacterial isolation and identification

The use of human specimens and all related experimental protocols were approved by the Committee on Human Research of the 307th Hospital of the People's Liberation Army and that of the First Affiliated Hospital of Anhui Medicial University, and carried out in accordance with the approved guidelines. The research involving biohazards and all related procedures were approved by the Biosafety Committee of the Beijing Institute of Microbiology and Epidemiology. Bacterial species was identified by 16S rRNA gene sequencing (Frank et al., 2008). The major plasmid-borne carbapenemase genes were screened for by PCR (Chen et al., 2015), followed by amplicon sequencing on ABI 3730 Sequencer.

### Plasmid conjugal transfer

Plasmid conjugal transfer experiments were carried out with the rifampin-resistant *Escherichia coli* EC600 (LacZ^−^, Nal^R^, Rif^R^) being used as recipient and strain 0801 or 7121 or 17285 or as donor (Chen et al., 2015). 3 ml of overnight culture of each of donor and recipient bacteria were mixed together, harvested and resuspended in 80 μl of Brain Heart Infusion (BHI) broth (BD Biosciences). The mixture was spotted on a 1 cm^2^ filter membrane that was placed on BHI agar (BD Biosciences) plate, and then incubated for mating at 37°C for 12–18 h. Bacteria were washed from filter membrane and spotted on Muller-Hinton (MH) agar (BD Biosciences) plate containing 1,000 μg/ml rifampin and 2 μg/ml imipenem for selection of *bla*_IMP_-positive *E. coli* transconjugants.

### Detection of carbapenemase activity

Activity of class A/B/D carbapenemases in bacterial cell extracts was determined via a modified CarbaNP test (Chen et al., 2015). Overnight bacterial cell culture in MH broth was diluted 1:100 into 3 ml of fresh MH broth, and bacteria were allowed to grow at 37°C with shaking at 200 rpm to reach an OD_600_ of 1.0 to 1.4. If required, ampicillin was used at 200 μg/ml. Bacterial cells were harvested from 2 ml of the above culture, and washed twice with 20 mM Tris-HCl (pH 7.8). Cell pellets were resuspended in 500 μl of 20 mM Tris-HCl (pH 7.8), and lysed by soniation, followed by centrifugation at 10,000 × g at 4°C for 5 min. 50 μl of the supernatant (the enzymatic bacterial suspension) were mixed with 50 μl of substrate I to V, respectively, followed by incubation at 37°C for a maximum of 2 h. Substrate I: 0.054% phenol red plus 0.1 mM ZnSO_4_ (pH7.8). Substrate II: 0.054% phenol red plus 0.1 mM ZnSO_4_ (pH7.8), and 0.6 mg/μl imipenem. Substrate III: 0.054% phenol red plus 0.1 mM ZnSO_4_ (pH7.8), 0.6 mg/μl mg imipenem, and 0.8 mg/μl tazobactam. Substrate IV: 0.054% phenol red plus 0.1 mM ZnSO_4_ (pH7.8), 0.6 mg/μl mg imipenem, and 3 mM EDTA (pH7.8). Substrate V: 0.054% phenol red plus 0.1 mM ZnSO_4_ (pH7.8), 0.6 mg/μl mg imipenem, 0.8 mg/μl tazobactam, and 3 mM EDTA (pH7.8).

### Bacterial antimicrobial susceptibility test

Bacterial antimicrobial susceptibility was tested by BioMérieux VITEK 2 and interpreted as per Clinical and Laboratory Standards Institute (CLSI) guidelines (ClSI, 2015).

### Plasmid sequencing and sequence assembly

Plasmid DNA was isolated from *E. coli* transconjugant using a Qiagen Large Construct Kit, and then sequenced with a paired-end library with an average insert size of 500 bp and a mate-pair library with average insert size of 5,000 bp, using an Illumina MiSeq sequencer (Illumina). Reads from each sample were trimmed to remove poor quality sequences, and then the contigs were assembled with Newbler 2.6 (Nederbragt, 2014).

### Sequence annotation and genome comparison

Open reading frames and pseudogenes were predicted using RAST 2.0 (Brettin et al., 2015) combined with BLASTP/BLASTN searches (Boratyn et al., 2013) against the UniProtKB/Swiss-Prot (Boutet et al., 2016) and RefSeq (O'leary et al., 2016) databases. Annotation of resistance genes, mobile elements, and other features was carried out using the online databases including CARD (Jia et al., 2016), ResFinder (Zankari et al., 2012), BacMet (Pal et al., 2014), ISfinder (Siguier et al., 2006), INTEGRALL (Moura et al., 2009), and the Tn Number Registry (Roberts et al., 2008). Multiple and pairwise sequence comparisons were performed using MUSCLE 3.8.31 (Edgar, 2004) and BLASTN, respectively. Gene organization diagrams were drawn in Inkscape 0.48.1.

### Nucleotide sequence accession numbers

The complete sequences of p0801-IMP, p7121-IMP, and p17285-IMP were submitted to GenBank under accession numbers KT345947, KX784502, and KX784503, respectively.

## Results

### Case reports

*K. pneumoniae* 0801, *K. oxytoca* 7121, and *C. freundii* 17285 were isolated from three different inpatients designated Patient 1 to Patient 3, respectively, with nosocomial infections. Patient 1 was a 35-year-old woman admitted to Hospital 1 in May 2013 and diagnosed to have acute lymphoblastic leukemia, and she received chemotherapy for 1 week. Pulmonary infection, septicemia and recurrent fever occurred during chemotherapy, and she received empirical intravenous administration with moxifloxacin. *K. pneumoniae* 0801was isolated from the blood specimens on the next day after chemotherapy. The patient was discharged 3 days later upon request from her family members.

Patient 2 was a 43-year-old woman admitted to Hospital 1 in January 2014 and diagnosed to have acute myeloid leukemia, and she received hematopoietic stem cell transplantation. Pulmonary infection occurred in the convalescent period, and *K. oxytoca* 7121 was then isolated from the sputum specimens. The patient received intravenous administration with flucloxacillin empirically at first, which was switched into levofloxacin based on antimicrobial susceptibility test results. Her symptoms associated with pulmonary infections progressively improved. The patient was discharged at 2 weeks after transplantation.

Patient 3 was a 66-year-old woman admitted to Hospital 2 in July 2013 and diagnosed to have adult onset Still's disease, and she received antianaphylactic treatment. Urinary tract infection occurred at 1 week after hospitalization, and *C. freundii* 17285 was then isolated from the voided midstream urine specimens. The patient received intravenous administration with teicoplanin. Her symptoms associated with infection and adult onset Still's disease progressively improved. The patient was discharged at 2 weeks after hospitalization.

### General features of resistant strains

PCR screening assay indicated the presence of *bla*_IMP_ but not any of the other carbapenemase genes tested in strains 0801, 7121, and 17285, and the *bla*_IMP_-carrying plasmids were designated p0801-IMP, p7121-IMP, and p17285-IMP, respectively. Each of these plasmids could be transferred into strain EC600 through conjugation, generating *E. coli* transconjugants 0801-IMP-EC600, 7121-IMP-EC600, and 17285-IMP-EC600, respectively, indicating that all these three plasmids are conjugative. All the above wild-type and transconjugant strains have the class B carbapenemase activity (data not shown), and are resistant to ceftriaxone, ceftazidime, imipenem and meropenem (Table 1). Strains 0801, 7121, and 17285 are resistant to gentamicin but the other strains remain susceptible to this drug, and all of them are susceptible to amikacin.

**Table 1:** **Antimicrobial drug susceptibility profiles**.

**Category**	**Antibiotics**	**MIC (mg/L)/antimicrobial susceptibility**						
		**0801**	**0801-IMP-EC600**	**7121**	**7121-IMP-EC600**	**17285**	**17285-IMP-EC600**	**EC600**
Third-generation cephalosporins	Ceftriaxone	>=64/R	>=64/R	>=64/R	>=64/R	>=64/R	>=64/R	<=1/S
	Ceftazidime	>=64/R	>=64/R	>=64/R	>=64/R	>=64/R	>=64/R	<=1/S
Carbapenems	Imipenem	>=16/R	>=16/R	>=16/R	>=16/R	>=16/R	>=16/R	<=1/S
	Meropenem	4/R	8/R	4/R	4/R	>=16/R	4/R	<=0.25/S
Aminoglycosides	Gentamicin	>=16/R	2/S	>=16/R	<=1/S	>=16/R	2/S	<=1/S
	Amikacin	<=2/S	<=2/S	<=2/S	<=2/S	<=2/S	<=2/S	<=2/S
Fluoroquinolones	Ciprofloxacin	<=0.25/S	<=0.25/S	<=0.25/S	<=0.25/S	>=4/R	<=0.25/S	<=0.25/S
	Levofloxacin	1/S	0.5/S	<=0.25/S	0.5/S	>=8/R	0.5/S	0.5/S

*S, sensitive; R, resistant*.

### Overview of sequenced plasmids

**Figure 1:**
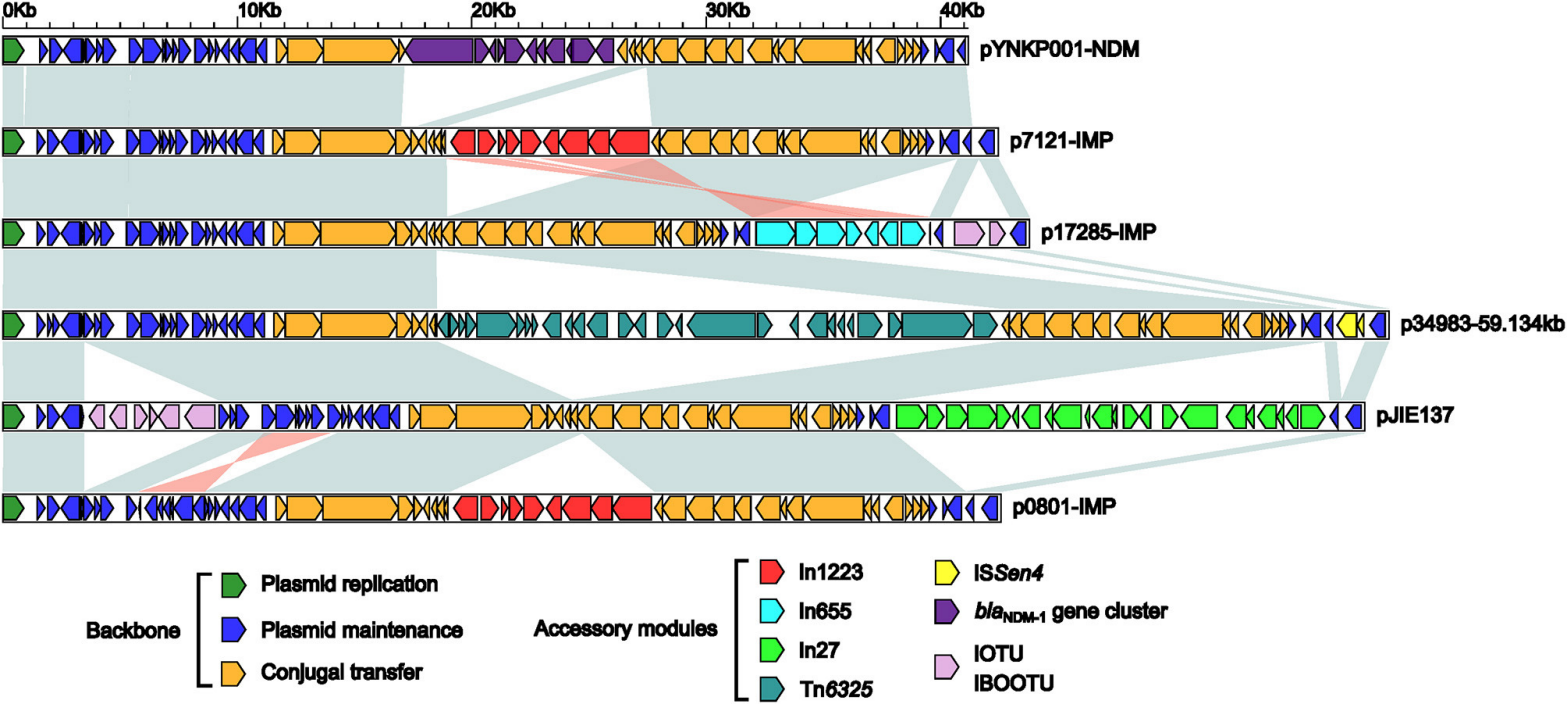
**Linear comparison of sequenced plasmids**. Genes are denoted by arrows. Genes, mobile elements and other features are colored based on function classification. Shading denotes regions of homology (>95% nucleotide identity).

Genome sequencing shows that p0801-IMP, p7121-IMP, and p17285-IMP have 42,580-bp, 42,461-bp and 43797-bp circularly closed DNA sequences, respectively, all of which carry 54 predicted open reading frames in total (Figure S1). These three plasmids belong to the IncN2 group because each of them contains an IncN2-type *repA* (plasmid replication initiation) gene. Further comparative genomics analysis is applied to all the five integron-carrying IncN2 plasmids p0801-IMP, p7121-IMP and p17285-IMP (this study), pJIE137 (Partridge et al., 2012), and p34983-59.134kb (accession number CP010378), together with the IncN2 reference plasmid pYNKP001-NDM (Sun et al., 2015), and the modular structure of each plasmid is divided into the IncN2 backbone as well as one or more accessory modules inserted at different sites of the backbone (Figure S1, Figure 1). Although p271A is the first fully sequenced IncN2 plasmid, pYNKP001-NDM is more appropriate as the IncN2 reference, because a 5.2-kb backbone region within the CUP-controlled regulon is absent from p271A relative to pYNKP001-NDM (Poirel et al., 2011; Sun et al., 2015).

### Backbones of integron-carrying IncN2 plasmids

The six plasmids involved in genomic comparison possess conserved IncN2 backbones, each of which can be further divided into the regions responsible for plasmid replication (*repA* and its iterons), maintenance [the CUP (conserved upstream repeat)-controlled regulon, the *stbABC*-*orfD* operon, and *resD*), and conjugal transfer (*tra* and *kikA*-*korB*) (Figure S1, Figure 1). There are two major differences among the backbones of these six plasmids: (i) a region between *repA* and its iterons from pYNKP001-NDM differs from all the other counterparts, and (ii) insertion, deletion, and rearrangement occur within the CUP-controlled regulons.

**Figure 2:**
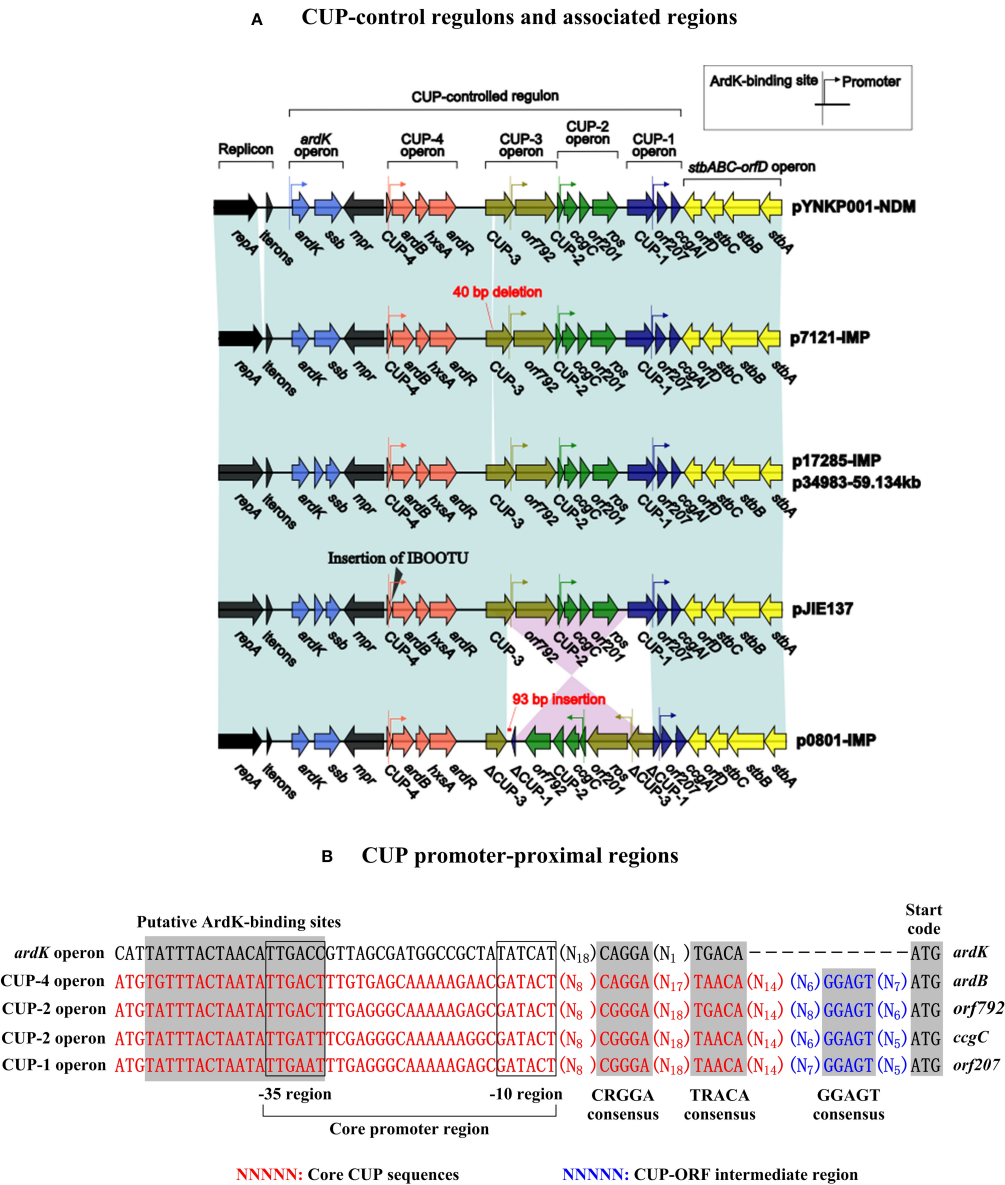
**CUP-related sequences. (A)** CUP-control regulons and associated regions. Genes are denoted by arrows. Genes, mobile elements and other features are colored based on function classification. Shading denotes regions of homology (>95% nucleotide identity). **(B)** CUP promoter-proximal regions. Shown are putative ArdK-binding sites, core promoter −10 and −35 regions, and 3 different consensus sequences within CUP promoter-proximal regions.

Gene organization and function of the IncN1 CUP-controlled regulon (Delver and Belogurov, 1997) have been described in the IncN1 reference plasmid R46 (accession number AY046276). Similarly, four putative operons (i.e., the *ardK* operon, the CUP-4 operon, the CUP-3 operon, the CUP-2 operon and the CUP-1 operon) arranged in the same orientation are annotated within the IncN2 CUP-controlled regulon, and CUP-4, CUP-3, CUP-2 and CUP-1 are located at the 5′-ends of the last four operons, respectively (Figure 2). Each of these operons contains a putative ArdK-binding site and a promoter, accounting for ArdK-dependent expression of operon-borne genes. A 40-bp deletion is found within CUP-3 of p7121-IMP. An IS*Ecp1*-*bla*_CTX-*M*-62_-Δ*orf477*-*orfRA1-14* transposition unit is inserted between CUP-4 and *ardB* in pJIE137 (Partridge et al., 2012), which would impair the gene expression of the CUP-4 operon. In p0801-IMP, homologous recombination medicated by CUP-3 and CUP-1 likely leads to an inversion of the *orf792* to *ros* region as well as the disruption of CUP-3 and CUP-1.

### Accessory regions of integron-carrying IncN2 plasmids

**Figure 3:**
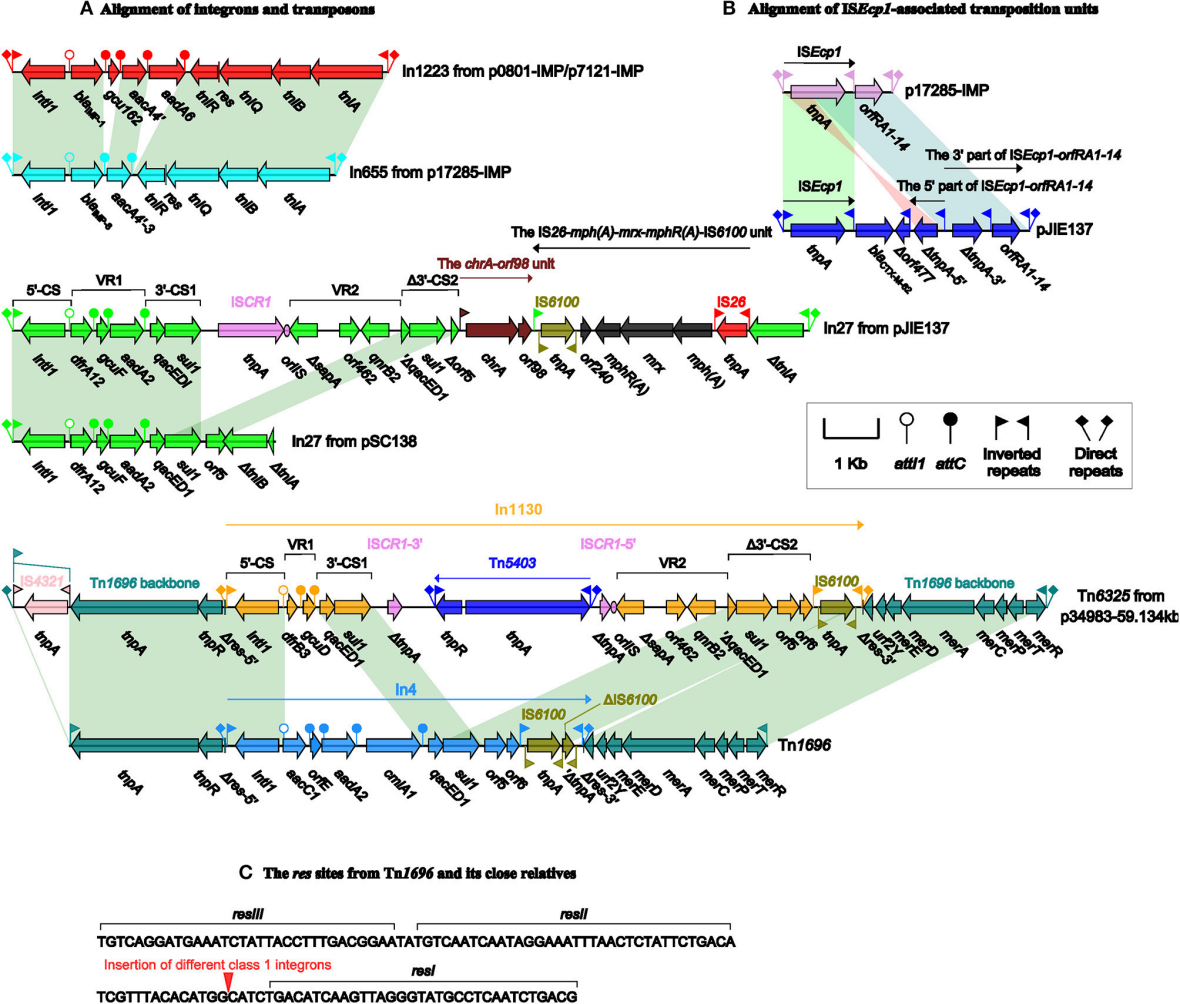
**Plasmid accessory modules**. Genes are denoted by arrows. Genes, mobile elements and other features are colored based on function classification. Shading denotes regions of homology (>95% nucleotide identity). Shown are the alignment of intergrons and transpsons **(A)** and IS*Ecp1*-associated transposition units **(B)**, and also the organization of the *res* sites from Tn*1696* and its derivatives **(C)**.

p0801-IMP and p7121-IMP carry a novel class 1 integron In1223, containing 5′-CS, the cassette array *bla*_IMP-1_-*gcu162*-*aacA4*-*aadA6*, and the complete Tn*402 tni* module (*tniABQ-res-tniR)*, which is bordered by IRi (inverted repeat at the integrase end) and IRt (inverted repeat at the *tni* end) (Figure 3A). *bla*_IMP-1_ and *aacA4*/*aadA6* account for resistance to carbapenems and aminoglycosides, respectively, while *gcu162* is a novel gene cassette of unknown function.

There are two accessory modules in each of pJIE137 (Partridge et al., 2012) and p17285-IMP. As shown in Figures 3A,B, p17285-IMP contains In655 (inserted into *resD*) and a 2554-bp IS*Ecp1*-*orfRA1-14* transposition unit (inserted between *orf333* and *orf648*), while pJIE137 harbors IS*Ecp1*-*bla*_CTX-*M*-62_-Δ*orf477*-*orfRA1-14* (inserted within the CUP-4 operon) and In27 (inserted between *resD* and *orf333*). In655 differs from In1223 by presence of a distinct cassette array *bla*_IMP-8_-*aacA4* and, notably, its Tn*402*-family *tni* module is maximally only 95% identical to the others at nucleotide level. IS*Ecp1* captures and arranges *orfRA1-14* and *bla*_CTX-*M*-62_-Δ*orf477* at its immediately downstream, which generates the transposition units IS*Ecp1*-*orfRA1-14* and IS*Ecp1*-*bla*_CTX-*M*-62_-Δ*orf477*, respectively, bordered by terminal inverted repeats IRL_*ISEcp1*_ and IRR_*ISEcp1*_. Similar IS*Ecp1*-*bla*_CTX-*M*_-Δ*orf477* structures (containing different variants of the *bla*_CTX-*M*-1_ group) are found on plasmids from various bacterial hosts, while the IS*Ecp1*-*orfRA1-14* elements are found on plasmids from only enterobacterial species. IS*Ecp1*-*bla*_CTX-*M*-62_-Δ*orf477*-*orfRA1-14* is a hybrid of IS*Ecp1*-*bla*_CTX-*M*-62_-Δ*orf477* and an IS*Ecp1*-*orfRA1-14*-related element that originates from splitting of IS*Ecp1*-*orfRA1-14* into the 5′- and 3′-parts, followed by inversion of the 5′-part (Zong et al., 2010).

Tn*6325* (Figure 3A) from p34983-59.134kb is a novel derivative of Tn*1696* belonging to the Tn*21* subgroup of the Tn*3* transposon family. Tn*1696*, located in the IncP1 plasmid R1033 from clinical *P. aeruginosa*, is generated from insertion of In4 within the resolution (*res*) site of a transposon backbone structure IRL-*tnpA* (transposase)-*tnpR* (resolvase)-*res*-*mer* (mercury resistance locus)-IRR, interrupting *res* into two separate parts (Partridge et al., 2001). Close Tn*1696* relatives, which contain different In4-type integrons inserted at exactly the same position as In4, have been found on plasmids such as pHCM1, pSRC125 and pSRC26 (Cain et al., 2010). Tn*6325* differs from Tn*1696* by (i) insertion a distinct integron In1130 at the same position as In4 within *res* (Figure 3C), and (ii) disruption of IRL_Tn6325_ into two parts by IS*4321* (Figure 3A) that is a hunter of terminal inverted repeats of Tn*21* subgroup transposons (Partridge and Hall, 2003).

The modular structure of a typical complex class 1 integron is organized sequentially as IRi, 5′-CS, variable region 1 (VR1), the first copy of 3′-CS [3′-CS1: *qacED1* (quaternary ammonium compound resistance)-*sul1* (sulfonamide resistance)], the common region IS*CR1*, VR2, the second copy of 3′-CS (3′-CS2; *qacED1*-*sul1*-*orf5-orf6*), *tni*, and IRt (Partridge et al., 2009). In27 from pJIE137 and In1130 from p34983-59.134kb (Figure 3A) belong to complex class 1 integrons because they contain all of the above core components with modifications of 3′-CS2. For both In27 and In1130, the connection of VR2 [Δ*sapA-orf462-qnrB2* (quinolone resistance)] with 3′-CS2 leads to the truncation of *qacED1* at the 5′-terminus of 3′-CS2. In addition, the *tni* module within the 3′-CS2 has been replaced by an IS*6100* element in In1130, while the 3′-CS2 of In27 is interrupted into two separate parts Δ*qacED1*-*sul1*-Δ*orf5* and Δ*tniA* due to the insertion of a 6.8-kb region [composed of the chromate-resistance unit *chrA*-*orf98* and the macrolide-resistance unit IS*26*-*mph(A)*-*mrx*-*mphR(A)*-IS*6100*], which is highly similar to the 3′-region of In37 from p112298-KPC (Feng et al., 2015). In In1130, IS*CR1* is interrupted by the insertion of a cryptic Tn*3*-family unit transposon Tn*5403*.

The insertion of each of In1223, In655, IS*Ecp1*-*orfRA1-14*, In27, IS*Ecp1*-*bla*_CTX-*M*-62_- Δ*orf477*-*orfRA1-14*, In1130 and Tn*6325* into the relevant plasmids leaves target site duplication signals of transposition, manifesting as various types of 5-bp direct repeat at the sites of insertion.

## Discussion

**Figure 4:**
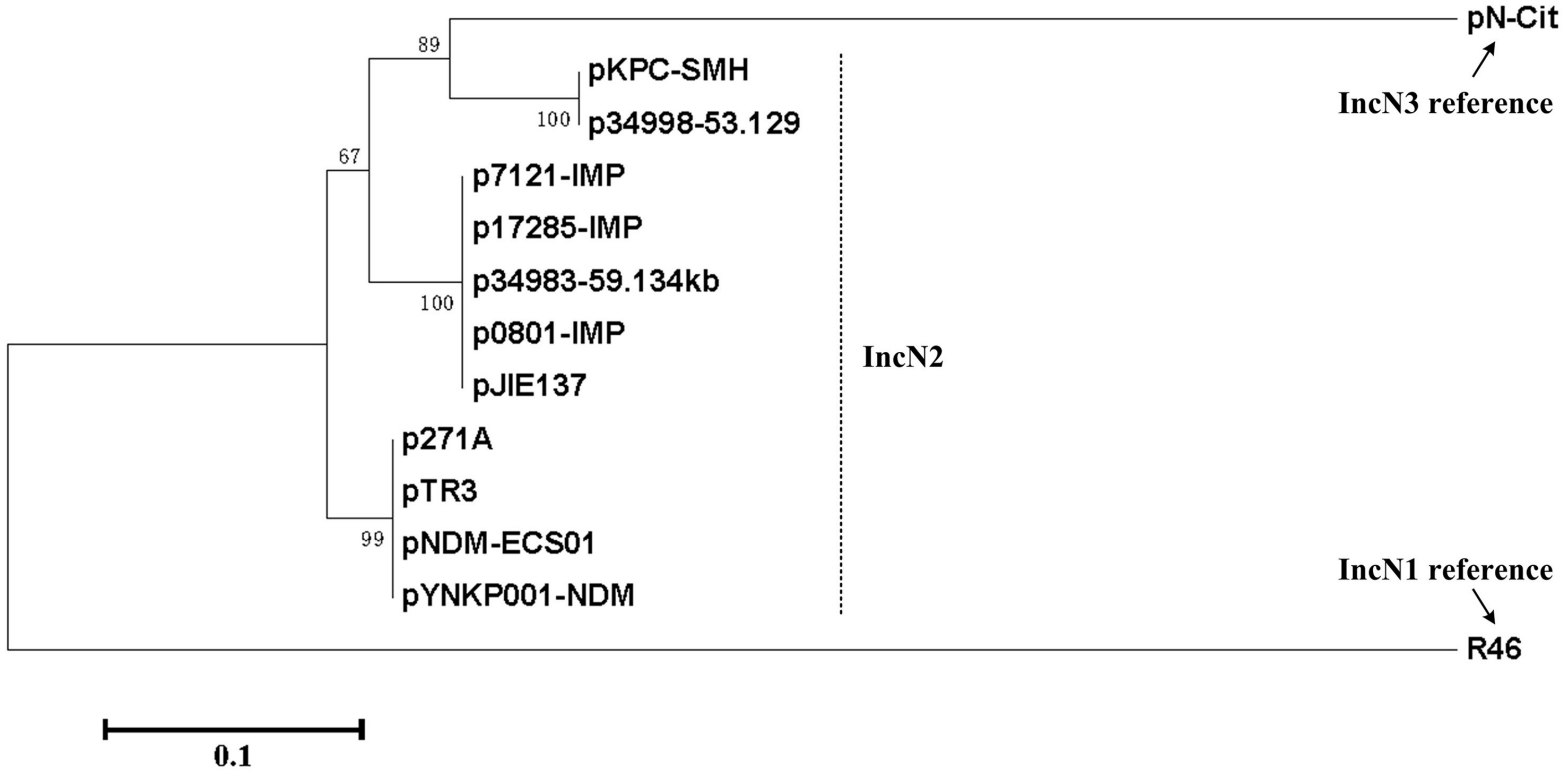
**Phylogenetic tree of *repA* sequences**. The nucleotide sequences of the *repA* coding regions from all the fully sequenced IncN2 plasmids together with R46 and pN-Cit (Villa et al., 2013) as the IncN1 and IncN3 reference, respectively, are aligned with MUSCLE 3.5 (Edgar, 2004). An unrooted neighbor-joining tree is inferred from the aligned sequences by using MEGA7 (Kumar et al., 2016) with calculation of evolutionary distances by the Maximum Composite Likelihood method. The percentages of replicate trees in which the associated taxa clustered together in the bootstrap test (500 replicates) are shown next to the branches.

A collection of fully sequenced plasmids including p0801-IMP, p7121-IMP, p17285-IMP, p271A, pYNKP001-NDM, pNDM-ECS01, pTR3, p34983-59.134kb, pJIE137, pKPC-SMH, and p34998-53.129kb (Poirel et al., 2011; Chen et al., 2012; Partridge et al., 2012; Netikul et al., 2014; Sun et al., 2015; Tijet et al., 2016) carry the IncN2 replicon and very similar backbones, which dramatically differ from IncN1 and IncN3, and thereby they are assigned into the IncN2 subgroup (Figure 4).

Each of the four class 1 integrons including In1223 from p0801-IMP/p7121-IMP, In655 from p17285-IMP, In27 from pJIE137, and In1130 from p34983-59.134kb has a complete set of IRi/IRt, *intI1*, and *attI1*. In1223/In27, In655, and In1130 have the promoters PcW_TGN-10_ (Strong) (Nesvera et al., 1998), PcS (Strong) (Collis and Hall, 1995), and PcW (weak) combined with P2 (strong) (Wei et al., 2011), respectively, which would drive the high-level expression of cassette-borne genes.

Tn*402* acts as a primary carrier of class 1 integrons, and the evolution of Tn*402*-associated class 1 integrons involves at least three stages as summarized previously: stage I, insertion of ancestral integron sequence (containing captured gene cassettes but lacking 3'-CS) into Tn*402* (harboring the *tni* module) to generate a hybrid structure, thereby combining the ability of integron to capture gene cassettes with the mobility of Tn*402*, which occurs prior to or concomitant with capture of *qacE*; stage II, capture of *sul1*-*orf5-orf6* and then formation of 3′-CS (*qacED1*-*sul1*-*orf5-orf6-tni*) after deletions between *qacE* and *sul1*; and stage III, deletions within the *tni* region, impairing the *tni*-mediated mobility (Chen et al., 2014). In1223 and In655 represent ancestral Tn*402*-associated integrons at stage I, while In27 from pSC138 (Chiu et al., 2005) and In4 from Tn*1696* are at stage III (Figure 3A). In27 from pJIE137 and In1130 from p34983-59.134kb have evolved into complex class 1 integron with integration of one or more additional regions containing several resistance markers, which might involve complex homologous recombination events involving IS*6100* and IS*26*(Feng et al., 2015).

The 7 mobile elements including In1223 from p0801-IMP/p7121-IMP, In655 and IS*Ecp1*-*orfRA1-14* from p17285-IMP, In27 and IS*Ecp1*-*bla*_CTX-*M*-62_-Δ*orf477*-*orfRA1-14* from pJIE137, and In1130 and Tn*6325* from p34983-59.134 kb are inserted at different sites and their mobilization into relevant plasmids leaves targeting signals of transposition, indicating that they are simple insertions due to transposition without adjacent deletions and rearrangements. The relatively small IncN2 backbones are able to integrate different mobile elements such as integrons, transposons and insertion sequence-based transposition units, which carry different resistance markers, thereby promoting accumulation and spread of antimicrobial resistance among bacterial species. Comparative genomics analysis of a larger collection of fully sequenced IncN1, IncN2, and IncN3 plasmids would promote us to gain deeper understanding of the horizontal transfer of antimicrobial resistance genes through mobile genetic elements as well as the molecular evolution mechanisms of diversification of IncN plasmid scaffolds. The combination of additional molecular epidemiological investigation will gain the highlights into not only the ability of plasmids to transmit among bacterial species and genera but also the underlying mechanisms of antibiotic resistance spread associated with hospitalized patients.

## Author contributions

DSZ, SC, and JW conceived the study and designed experimental procedures. XJ, ZY, XY, and HF, performed the experiments. XJ, DSZ, QS, and DFZ analyzed the data. XJ, YT, YX, JF, WC, and YS contributed reagents and materials. DSZ, SC, XJ, and JW wrote this manuscript.

## Consent statement

Written informed consent was obtained from all participants.

### Conflict of interest statement

The authors declare that the research was conducted in the absence of any commercial or financial relationships that could be construed as a potential conflict of interest.

## Supplementary material

Figure S1 **Schematic maps of sequenced plasmids**. Genes are denoted by arrows, and the backbone and accessory module regions are highlighted in black and color, respectively. The innermost circle presents GC-skew [(G−C)/(G+C)], with a window size of 500 bp and a step size of 20 bp. The next-to-innermost circle presents GC content.
