# Supplementary figures and images for: Sequencing of blaIMP-Carrying IncN2 Plasmids, and Comparative Genomics of IncN2 Plasmids Harboring Class 1 Integrons
Source: Front Cell Infect Microbiol. 2017 Mar 30;7:102. doi: 10.3389/fcimb.2017.00102 (PMC5371602; doi:10.3389/fcimb.2017.00102)

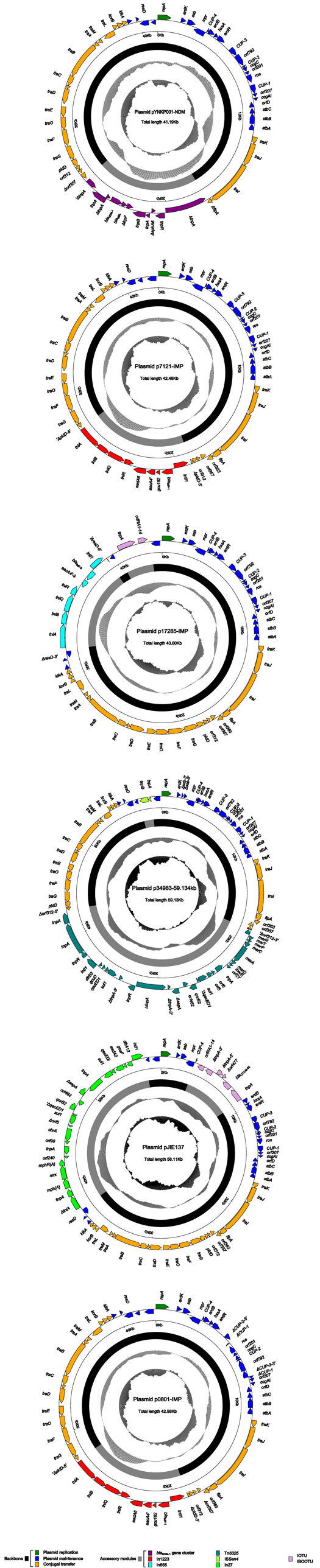

Supplement: Figure S1 — Schematic maps of sequenced plasmids. Genes are denoted by arrows, and the backbone and accessory module regions are highlighted in black and color, respectively. The innermost circle presents GC-skew [(G−C)/(G+C)], with a window size of 500 bp and a step size of 20 bp. The next-to-innermost circle presents GC content. [file Image1.TIF]
